# Post-progression treatment in cancer randomized trials: a cross-sectional study of trials leading to FDA approval and published trials between 2018 and 2020

**DOI:** 10.1186/s12885-023-10917-z

**Published:** 2023-05-17

**Authors:** Timothée Olivier, Alyson Haslam, Vinay Prasad

**Affiliations:** 1 Department of Oncology, Geneva University Hospital, 4 Gabrielle-Perret-Gentil Street, Geneva, 1205 Switzerland; 2 Department of Epidemiology and Biostatistics, University of California San Francisco, 550 16th St, 2nd Fl, San Francisco, CA 94158 USA

**Keywords:** Post-progression therapy, Randomized controlled trials, Global oncology, Drug regulation, Cross-over

## Abstract

**Background:**

Suboptimal treatment upon progression may affect overall survival (OS) results in oncology randomized controlled trials (RCTs). We aim to assess the proportion of trials reporting post-progression treatment.

**Methods:**

This cross-sectional analysis included two concurrent analyses. The first one examined all published RCTs of anti-cancer drugs in six high impact medical/oncology journals between January 2018 and December 2020. The second studied all US Food and Drug Administration (FDA) approved anti-cancer drugs during the same period. Included trials needed to study an anti-cancer drug in the advanced or metastatic setting. Data abstracted included the tumor type, characteristics of trials, and reporting and assessment of post-progression treatment.

**Results:**

There were 275 published trials and 77 US FDA registration trials meeting inclusion criteria. Assessable post-progression data were reported in 100/275 publications (36.4%) and 37/77 approvals (48.1%). Treatment was considered substandard in 55 publications (*n* = 55/100, 55.0%) and 28 approvals (*n* = 28/37, 75.7%). Among trials with assessable post-progression data and positive OS results, a subgroup analysis identified substandard post-progression treatment in 29 publications (*n* = 29/42, 69.0%) and 20 approvals (*n* = 20/26, 76.9%). Overall, 16.4% of publications (45/275) and 11.7% of registration trials (9/77) had available post-progression data assessed as appropriate.

**Conclusion:**

We found that most anti-cancer RCTs do not report assessable post-progression treatment. When reported, post-progression treatment was substandard in most trials. In trials reporting positive OS results and with assessable post-progression data, the proportion of trials with subpar post-progression treatment was even higher. Discrepancies between post-progression therapy in trials and the standard of care can limit RCT results’ applicability. Regulatory rules should enforce higher requirements regarding post-progression treatment access and reporting.

**Supplementary Information:**

The online version contains supplementary material available at 10.1186/s12885-023-10917-z.

## Background

There are two broad classes of randomized trials in cancer medicine. The first seeks to establish fundamental efficacy, which tests novel compounds that have hitherto not been used in that tumor type. If these trials seek to show new drugs improve patient-centered outcomes, patients in both arms should receive the best available standard-of-care (SOC) upon progression.

Having demonstrated fundamental efficacy, the second class of trials seeks to establish the optimal sequence. Cancer treatment often consists of consecutive therapies: first-, second-, subsequent “lines”. After one line of treatment, the next line is usually initiated upon progression. The goal is to determine how to maximize survival and quality-of-life (QoL) while using the least amount of drugs (minimizing toxicity, financial and time burdens).

When we interpret either class of trials (testing fundamental efficacy or optimal sequence), post-progression treatment is key [1–3]. Post-progression data and reporting have been described in multiple myeloma and renal cell carcinoma [4, 5]. Here we sought to broadly appraise the quality of post-progression therapy in trials of all tumor types. We assessed and characterized the reporting of post-progression treatment in trials published in top journals and in FDA registration trials. We also proposed a framework to appraise post-progression treatment.

## Methods

### Study design and research strategy

This was a retrospective, cross-sectional study that sought anti-cancer drug randomized controlled trials (RCTs) published in six high impact medical and oncology journals, as well as RCTs that led to FDA approval of an anti-cancer drug. We adhered to Strengthening the Reporting of Observational studies in Epidemiology (STROBE) reporting guidelines.

### Inclusion and exclusion criteria

Selected trials needed to (1) be a comparative RCT; (2) study an anti-cancer drug (surgical or radiotherapeutic were included only if there was a difference in an anti-cancer drug assignment between arms); (3) be in the advanced or metastatic setting; (4) and have performed the analysis in the originally randomized groups. The exclusion criteria were (1) trials not evaluating direct anti-cancer interventions; (2) studies that combined multiple RCTs; and (3) subset analysis of a previous study, cost-effectiveness analysis, or biomarker exploring studies. Due to their unique biological, clinical, ethical and regulatory specificities, pediatric trials were excluded.

Searches were performed on July 27, 2021. Because we used publicly available data and this is not human subjects research, in accordance with 45 CFR §46.102(f), we did not submit this study to an institutional review board or require informed consent.

### Published articles and FDA approvals identification

As clinical practices not only depend on the novel registration of drugs (approvals), but also on trial results published in high-impact factor journals, we have decided to conduct analyses for both reporting types. Method S1 and Method S2 detail the trials identification methods.

### Data abstraction

Information abstracted for each trial included date of publication / approval; journal name; NCT number; tumor type; hematologic or non-hematologic cancer; setting (line of therapy); whether the trial tested the fundamental efficacy or the best sequence of a drug; design (open or blinded); trial phase; sponsor type (any industry involvement or not); whether post-progression was reported (yes, no, or present but not assessable); if reported, whether post-progression treatment in the control arm was appropriate as compared with SOC (yes or no); and if positive and statistically significant overall survival (OS) trial results were reported (yes or no). Because FDA approvals were mostly based on industry sponsored trials, study funding was not abstracted for approvals. For FDA registration trials, other publications were identified through the NCT number, and when multiple publications of the same trial were identified, we abstracted the more mature data.

In trials with assessable post-progression data, we classified trials into two categories. First, trials testing the fundamental efficacy of a drug are those that test a compound for the first time, usually in later lines. In these cases, the experimental drug should not be administered at progression in the control arm because the fundamental efficacy of the drug has not been established. Second, in trials testing the optimal sequence, we are assessing drugs (or drug classes) already proven to be beneficial. These trials may test a drug upfront where the drug is already SOC in second-line. This classification was determined to test, as an exploratory endpoint, whether these classifications were associated with differences in the type of post-progression data.

Two of the authors (A. H., T.O.) independently reviewed and abstracted data from each trial. A third reviewer (V.P.) adjudicated any discrepancies.

### Definitions: post-progression, post-protocol, and cross-over

There are overlapping definitions regarding cross-over, post-progression, and post-protocol therapies. For the purpose of our study, “post-progression” is defined by the treatment that a patient receives when disease progression occurs, regardless of the drug and regardless of whether it is within a trial protocol. “Cross-over”, meaning that the patient in the control arm receives the experimental arm at progression, is often restricted to the experimental drug only, is specified by the protocol, and occurs within the trial. It is, therefore, one specific subset of post-progression therapy. Lastly, “post-protocol” is often interchangeably used for “post-progression” treatment, but as post-progression therapy can occur during the protocol, we preferred to use “post-progression”.  Also, we assessed for post-progression therapy referring to systemic treatment (not radiotherapy nor surgery for instance).

### Post-progression assessment in the control arm and pre-specified rules

Because we assessed post-progression therapy in context of the SOC in high-income countries, the SOC was set according to FDA drugs approved in the respective setting during the time of trial enrolment. This was based on the assumption that the FDA approval date allowed the drug to be marketed in the USA. In some situations, the uptake may not be based on FDA approvals. For example, uptake can occur, based on National Comprehensive Center Network guidelines instead, or uptake can occur for several years without any formal recommendations. This was the case for checkpoint inhibitors in malignant mesothelioma [6]. In that regard, our rule was deemed conservative.

We pre-specified, to allow for reproducibility, 3 rules for determining whether systemic post-progression treatment was appropriate as compared with the SOC. The following applies to the control arm:

- Rule 1: among patients receiving post-progression therapy, no more than 10% of them should be deprived of a preferred approved therapy, including drugs with a similar mechanism of action. Based on our clinical experience (TO and VP), we considered it unlikely that, with a fair access to all treatment options, more than 10% patients could have a contra-indication to a drug with better efficacy or tolerance and receive a cytotoxic chemotherapy instead. If this happened, it was more likely due to a restricted access to the best available therapies (Fig S1).
- Rule 2: among patients receiving post-progression therapy, a maximum of 10% of them should crossover to the experimental drug or receive it as part of post-progression treatment when the drug is still a therapy with unproven efficacy in subsequent lines. Such use of crossover can be detrimental. We chose a 10% threshold, but it should ideally be restricted to the minimum (Fig S2).
- Rule 3: the proportion of patients receiving post-progression therapy, in comparable settings, should be at least 10 percentage-points higher in trials than in the real-world setting (Fig S3). This is because multiple studies show trial patients are younger, more fit, and more able to tolerate therapies [7, 8]. To assess this specific rule, we looked for real-world published data that needed to be peer-reviewed and published after 2015. The search was performed using PubMed.gov using the words “real-world”, “real-life”, and other keywords relevant to the specific setting. This rule may be relevant for both arms, an example is provided in the discussion.

Assessment was conducted independently by two reviewers (TO and VP) and final agreement was based on consensus. Each trial was coded with one rule (rule 1 was assessed before rule 2, and then rule 3). A trial which did not meet the criteria for at least one of the 3 rules was assessed as substandard.

### Statistical analysis

Frequencies were calculated for categorical variables. A Chi-squared test of independence was used to assess categorical differences in study qualities between 1) those reporting post-progression treatment and those that did not, and 2) those reporting appropriate treatment and those that did not. Statistical analyses were done using R version 3.6.2 (R Project for Statistical Computing) and a 2-tailed *P* value less than 0.05 was used for determining significance.

## Results

### Published trials

**Fig. 1:**
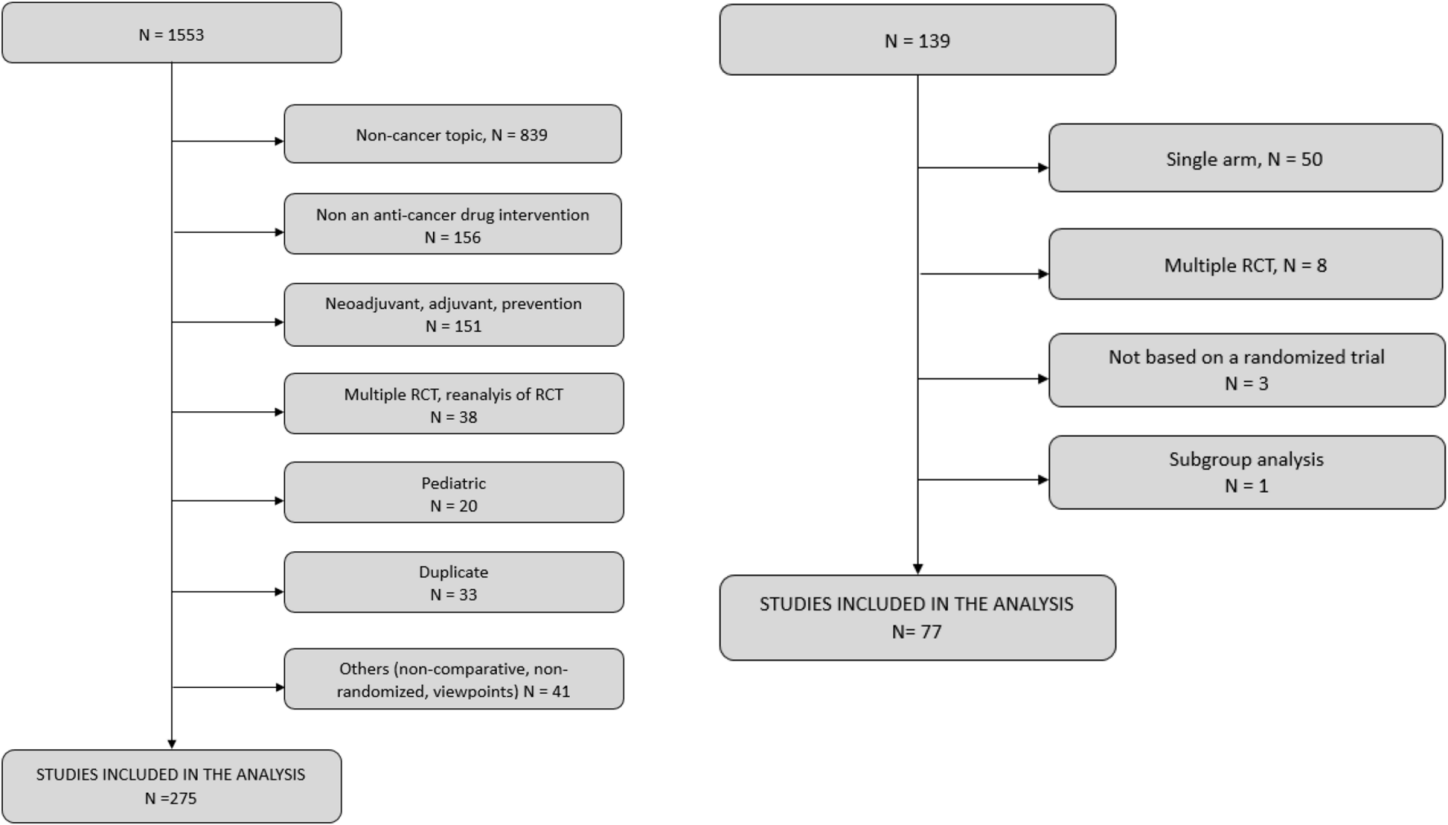
Flow charts of the top journal publications selection process (left panel), and of the FDA registration trials selection process (right panel)

There were 1553 articles reviewed, of which 275 met inclusion criteria (Fig. 1).

Among eligible studies, 89 studies (32.4%) were published in the *JCO,* 79 (28.7%) in *Lancet Oncology*, 53 (19.3%) in *NEJM*, 25 (9.1%) in *JAMA Oncology*, 26 (9.5%) in *Lancet*, and three studies (1.1%) in *JAMA*. Study qualities are described in Table 1. Details on tumor type are described in the Table S1. Post-progression data were assessable and reported in 100 articles (36.4%) and not reported in 166 articles (60.4%). Nine articles (3.3%) reported post-progression data with immature or too little information to assess for their adequacy and were coded as “not assessable”.

**Table 1:** Characteristics of selected published trials (*N* = 275) and trials leading to an FDA approval (*N* = 77), according to the availability of assessable post-progression data

	**PUBLISHED TRIALS**			**TRIALS LEADING TO AN FDA APPROVAL**		
	Total	Post-Progression data ^a^ **NO**	Post-Progression data **YES**	Total	Post-Progression data ^a^ **NO**	Post-Progression data **YES**
	275	175 (63.6%)	100 (36.4%)	77	40 (51.9%)	37 (48.1%)
**Sponsor:**						
Any industry involvment	237	143 (60.3%)	94 (39.6%)	NA ^b^	NA ^b^	NA ^b^
No industry involvment	38	32 (84.2%)	6 (15.8%)	NA ^b^	NA ^b^	NA ^b^
**Phase** ^c^						
≤ 2	71	58 (81.7%)	13 (18.3%)	4	4 (100%)	0 (0%)
≥ 3	204	117 (57.4%)	87 (42.6%)	73	36 (49.3%)	37 (50.7%)
**Design**						
Blind	91	52 (57.1%)	39 (42.9%)	31	15 (48.4%)	16 (51.6%)
Open	184	123 (66.8%)	61 (33.2%)	46	25 (54.3%)	21 (45.7%)
**Tumor Type**						
Hematologic malignancy	64	54 (84.4%)	10 (15.6%)	18	15 (83.3%)	3 (16.7%)
Solid Tumor	211	121 (57.3%)	90 (42.7%)	59	25 (42.4%)	34 (57.6%)
**OS Results**						
Positive	71	29 (40.8%)	42 (59.2%)	36	10 (27.8%)	26 (72.2%)
Others ^d^	204	146 (71.6%)	58 (28.4%)	41	30 (73.2%)	11 (26.8%)
**Setting:**						
First line	128	74 (57.8%)	54 (42.2%)	39	15 (38.5%)	24 (61.5%)
Second line or subsequent	92	61 (66.3%)	31 (33.7%)	15	7 (46.7%)	8 (53.3%)
Third line or subsequent	14	8 (57.1%)	6 (42.9%)	7	6 (85.7%)	1 (14.3%)
Fourth or fifth or subsequent	2	2 (100%)	0	2	2 (100%)	0
Maintenance	22	16 (72.7%)	6 (27.3%)	7	4 (57.1%)	3 (42.9%)
Mixed	17	14 (82.4%)	3 (17.6%)	7	6 (85.7%)	1 (14.3%)

^a^ Among the reported post-protocol data, 9 published trials and 2 trials leading to an approval did not reported enough data to be assessed for adequacy

^b^ *NA* Not available

^c^ There were 6 phase 1/2 trials and 4 phase 3/4 or 4 in the published trials, all were randomized trials

^d^ Others = either not an endpoint, not positive, not mature, or a non-inferiority result

**Fig. 2:**
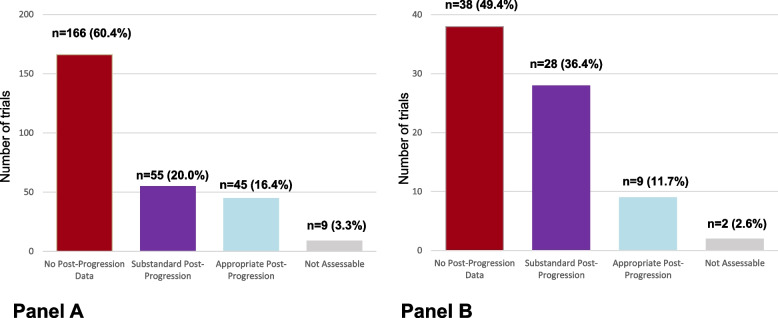
Post-progression data in anti-cancer randomized trials, in the advanced or metastatic settings. Legend: Panel **A** Published in 6 high impact journals between 2018 and 2020 (*N* = 275). Panel **B** Trials leading to an FDA approval between 2018 and 2020 (*N* = 77)

Post-progression treatment was assessed as appropriate in 45 articles (45/100 or 45.0% of trials reporting assessable post-progression data) and substandard in 55 published trials (55/100 or 55.0% of trials reporting assessable post-progression data; Fig. 2A). In trials with subpar post-progression data (*n* = 55), the reason was a low access to a preferred option in 33 reports (33/55 or 60.0%, rule 1), a crossover to an unproven therapy in 9 reports (16.4%, rule 2), or a low access to any option (in both arms) in 13 reports (23.6%, rule 3).

Studies that reported assessable post-progression data were more likely than those not reporting post-progression data (including those reporting not assessable data) to report on a solid tumor (90.0% vs. 69.1%; *P* < 0.001); to be phase 3 trials (87.0% vs. 66.9%; *P* < 0.001); to report positive OS results (42.0% vs. 16.6%; *P* < 0.001), and be industry-sponsored (94.0% vs. 81.7%; *P* = 0.008); but they were not more likely to be open label (61.0% vs. 70.3%; *P* = 0.1496).

Overall, 45 out of the 275 included trials (16.4%) had post-progression treatment reported and assessed as appropriate.

### FDA registration trials

There were 139 FDA approvals reviewed, of which 77 met inclusion criteria (Fig. 1). Study qualities are described in Table 1, with tumor types described in Table S2. For each trial, other publications were identified based on the NCT number through clinicaltrials.gov, identifying 225 single publications. Data on post-progression were abstracted, when multiple reports were available, from the most mature publication. Post-progression data were assessable and reported in 37 trials (48.1%) and not reported in 38 (49.4%) articles. Two RCTs (2.6%) were coded as “not assessable”.

Post-progression treatment was assessed as appropriate in 9 trials (9/37 or 24.3% of trials reporting assessable post-progression data) and substandard in 28 (28/37 or 75.7%; Fig. 2B). In trials with subpar post-progression data (*n* = 28), the reason was a low access to a preferred option in 19 reports (19/28 or 67.9%, rule 1), a crossover to an unproven therapy in 4 reports (14.3%, rule 2), or a low access to any option (in both arms) in 5 reports (17.9%, rule 3).

Studies that reported assessable post-progression data were more likely than those not reporting post-progression data (including those reporting not assessable data) to report on a solid tumor (91.9% vs. 62.5%; *P* = 0.006) and report positive OS results (70.3% vs. 25.0%; *P* < 0.001); but they were not more likely to be open label or a phase 3 trial (data not shown).

Overall, 9 out of the 77 included trials (11.7%) had post-progression treatment reported and assessed as appropriate.

### Trials reporting a positive OS benefit

**Fig. 3:**
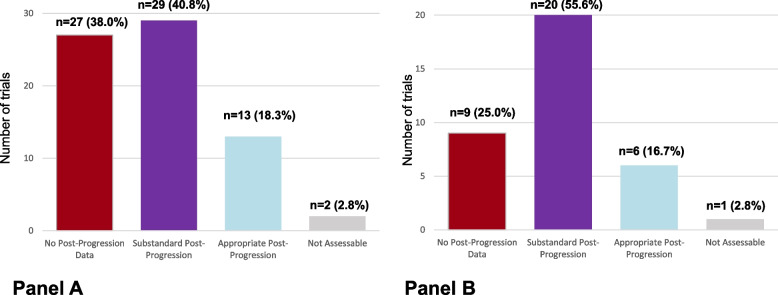
Subgroup analysis restricted to trials reporting positive overall survival results. Post-progression data in anti-cancer randomized trials, in the advanced or metastatic settings. Legend: Panel **A** Published in 6 high impact journals between 2018 and 2020 (*N* = 71). Panel **B** Trials leading to an FDA approval between 2018 and 2020 (*N* = 36)

There were 71 (71/275, 25.8%) publications and 36 FDA approvals (36/77, 46.8%) that reported positive OS results. Among them, assessable post-progression data were reported in 42 publications (42/71, 59.2%) and 26 approvals (26/36, 72.2%); no post-progression data in 27 publications (38.0%) and 9 approvals (25.0%); and data were not assessable in 2 publications (2.8%) and 1 approval (2.8%). In trials reporting assessable post-progression data, post-progression was assessed as appropriate in 13 publications (13/42 or 31.0%) and 6 approvals (6/26, 23.1%), and substandard in 29 publications (29/42, 69.0%) and 20 approvals (20/26, 76.9%) (Fig. 3A and B).

### Exploratory analysis

There were 64 trials that were included in both cohorts (the FDA registration trials and the published trials). After removing the duplicates, 104 single trials could be analyzed with assessable post-progression data. In this subset, we found that trials studying optimal sequence were more likely to limit access to a preferred option upon progression as compared to studies testing fundamental efficacy (chi-squared = 12.41, *p* = 0.006). The results of this analysis, including characteristics of crossover in trials’ design, are available in the Table S3. In trials with subpar post-progression data (*n* = 56), the reason was a low access to a preferred option in 34 reports (34/56 or 60.7%, rule 1), a crossover to an unproven therapy in 9 reports (16.1%, rule 2), or a low access to any option (in both arms) in 13 reports (23.2%, rule 3). Real-world data on which rule 3 was assessed are detailed in the Table S4. Additionally, in those trials (*n* = 104), the median percentage of patients receiving post-progression treatment in the control arm was 54.9% (SD = 18.8), which was statistically higher than the median for the experimental arm (45.0%, SD = 19.3, *p* = 0.001).

### Tumor types

The 3 most represented tumor types were non-small cell lung cancer, breast cancer, and prostate cancer (Tables S1 and 2). These tumor types are among the top 4 most prevalent cancers worldwide [9]. Examples of post-progression therapy in these tumor types (Discussion and Table 2) demonstrate the broad clinical significance of our findings.

**Table 2:** Types of post-progression therapy, with examples and description

**Type of post-progression therapy**	**Examples**	**Description**	**Desirable**
**Substandard post-progression therapy** **OR** **Low cross-over rate** **(to a therapy with proven efficacy in later lines)**	LATITUDE trial [10] - Abiraterone *versus* placebo in addition to ADT^a^ in castrate sensitive metastatic prostate cancer - Limited access to abiraterone at progression in the control arm, with 24% of patients treated upon progression receiving abiraterone	With substandard access to optimal post-progression treatment in the control arm (either within the protocol, or outside the protocol), this may favor the experimental arm	**NO**
**Standard post-progression therapy** **OR** **High cross-over rate** **(to a therapy with proven efficacy in later lines)**	PROFILE 1014 trial [11] - Crizotinib *versus* chemotherapy in first-line ALK-positive non-small cell lung cancer - Upon progression in the control arm, 98% of treated patients received a TKI		YES
**Crossover to a therapy with unproven efficacy in later lines** **(either within or outside the protocol)**	POLO-trial [12] - Olaparib versus placebo as maintenance after first-line chemotherapy in germline BRCA-mutated metastatic pancreatic cancer - 15% of patients received a PARP inhibitor upon progression in the control arm (with unproven efficacy at progression)	With crossover to a therapy with unproven efficacy, this may alter the correct interpretation of overall survival results (e.g. incorrectly attributing lack of survival benefit to crossover)	**NO**
**No crossover to a therapy with unproven efficacy in later lines** **(either within or outside the protocol)**	NGR015 trial [13] - NGR-hTNF *versus* placebo (plus best investigator choice) in second-line pleural mesothelioma - No patient received the investigational agent (NGR-hTNF) upon progression		YES
**Low access to any post-progression therapy**	MONALEESA-7 trial [14] - Ribociclib *versus* placebo (plus endocrine therapy) in mostly first-line hormone positive metastatic breast cancer - 73% received any therapy at progression in the control arm, 69% in the experimental arm, far less than in the real life	With limited access to any post-progression treatment in both arms (including the control arm), this may favor the experimental arm by limiting the “dilution” of benefit that could have occur with optimal access to standard subsequent lines	**NO**
**High access to any post-progression therapy**	FRENCH-LUNG-CANCER-GROUP [15] - Carboplatin plus etoposide *versus* topotecan in second-line small cell lung cancer - 63% of patients received a 3rd line		YES

^a^*ADT* Androgen-deprivation therapy

## Discussion

In this cross-sectional analysis, we found that post-progression treatment data were not available in 60% of publications and 49% of registration trials. Among trials with assessable post-progression data, the treatment was substandard in 55% of published trials and 76% of registration trials. We conducted a subgroup analysis in trials reporting statistically significant positive OS results and found that the proportion of post-progression therapy reporting was higher than in the overall cohort. Yet, when assessable, post-progression was appropriate in fewer trials than in the overall cohort.

Our findings show deficiency in the level of reporting and quality of subsequent treatment that patients may receive when enrolled in oncology RCTs. Similar findings have previously been reported in specific tumor types like in multiple myeloma [4] or renal cell carcinoma [5]. The need for conducting trials globally, which include middle and low-income countries with limited or no access to SOC, is one explanation for suboptimal post-progression treatment. However, this is not acceptable in our view, as these same trials are often the basis on which approvals, guidelines, and recommendations are implemented in high-income countries.

**Fig. 4:**
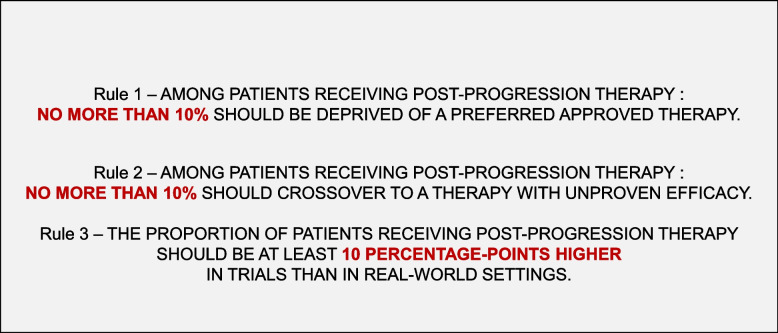
« Ten percent» pre-specified rules to assess post-progression treatment in the control arm of randomized clinical trials. Legend: post-progression therapy is referring to systemic treatment

Post-progression treatment access is indeed critical in appraising the results of RCT’s since substandard post-progression treatment may limit the generalizability of the reported results to places where optimal care is available. In line with a previous work [3], we identified three situations when undesirable post-progression treatment may occur (Fig. 4).

First, when a treatment is already approved in subsequent lines and tested upfront, the core question of the trial is if there is benefit  in moving the drug upfront. Free access to the treatment at progression in the control arm should therefore be mandatory. If not, the question whether upfront treatment is superior to the current standard, being treatment when the disease progresses, will remain unanswered (Fig S1). The LATITUDE trial randomized patients with metastatic castration-sensitive prostate cancer between androgen deprivation therapy alone or with abiraterone [10, 16]. In castration-resistant patients, abiraterone was SOC before the trial started enrolment. However, in LATITUDE, only 24% of control arm patients treated with a subsequent therapy received abiraterone [10]. As we previously highlight, “we cannot be sure that the survival advantage of early treatment would still exist if control patients had fair access to this drug” [1, 3].

Second, inappropriate use of crossover may confound a true verdict on the drug’s therapeutic effect. If a novel treatment is tested (with unproven efficacy in subsequent lines), patients in the control arm should not be offered this treatment at progression, and should not crossover. If the drug truly has a survival gain, crossover may obscure the benefit. Conversely, if the drug offers no survival gain, crossover may lead to a delay in the time when effective therapies will be provided. This may lead to superior OS in the experimental arm (not subject to crossover) being incorrectly imputed to better efficacy when it may be merely due to the fact that the intervention arm received superior post-progression therapy. The IMPACT study trial tested sipuleucel-T in patients with metastatic castration-resistant prostate cancer [17]. The crossover to sipuleucel-T in the control arm may have delayed access and lowered the proportion of patients in the control arm having access to life-prolonging therapies, possibly explaining the OS benefit with no PFS advantage (Fig S2) [3]. For these reasons crossover is not desirable in trials assessing the fundamental efficacy of products, and is discouraged by regulatory agencies [3, 18–20].

The last scenario is when the proportion of patients receiving post-progression treatment in the trial is inferior to real-world settings. Because of strict inclusion–exclusion criteria, trials are known to select patients with better performance status and fewer comorbidities [7]. It has been shown that 38% of patients in the real-world would be ineligible for trial participation [8]. As a consequence, because trial patients are generally healthier and more fit than average cancer patients, the proportion of patients receiving a subsequent therapy in trials should be higher than in real-life. If not, this limitation will affect both arms. However, we contend, as others do, that such a trial may favor the experimental arm and be more likely to conclude a survival benefit when the real-world use of the drug would not yield a similar result. Many researchers state that post-progression treatment can “dilute” the PFS or overall response rate advantage [21, 22]. The MONALEESA-7 trial tested ribociclib against placebo (plus hormonal therapy) in patients mostly in the first-line setting of hormone-sensitive advanced or metastatic breast cancer [23]. Post-progression treatment was low in both arms (73% in the control arm, 69% in the experimental arm) [14], when real-life data demonstrated higher access (up to 92% after first-line hormonal therapy) [24]. Would the same OS advantage have occurred if both arms had optimal access to subsequent therapeutic options? The dilution is precisely the question at hand: there is no reason to give the drug sooner, with the potential of more toxicity, if subsequent therapies achieve the same result (Fig S3).

Examples of desirable and undesirable scenarios are provided in Table 2. We also described the “ten percent” rules, which we prespecified and applied to assess post-progression treatment in our work (Fig. 4). Others may disagree, and they can propose and apply other rules, and we encourage this effort.

Assessment scores for the value of anti-cancer treatments, such as the ASCO-Value Framework Net Health Benefit and ESMO-MCBS, do not consider the quality of post-progression therapy. Similarly, the Cochrane Risk of Bias assessment (RoB2) does not systematically evaluate post-progression data. Yet, when analyzing results from clinical trials that have suboptimal post-progression therapy, it is crucial to be aware of these limitations in order to make informed clinical decisions. For instance, a trial with proper post-progression care will likely produce more trustworthy results compared to a trial with inadequate or unreported post-progression data. Ultimately, the lack of trials that accurately reflect standard practice is a significant issue. One potential solution to this problem is for regulatory bodies to only grant marketing authorization for trials that provide optimal post-progression therapy in both the experimental and control groups.

### Limitations

Our work has strengths and limitations. First, the time-period is limited to three years, and this is not a systematic review. Our aim was to capture recent trends in reporting post-progression therapy, and we encourage others to expand upon our work to cover a broader time period. However, our work is the first to encompass all tumor-types, and to conduct analyses in both FDA registration trials and top journal publications. Second, the prespecified rules can be questioned and are not supported by randomized data. We prespecified them to allow for reproducibility, and they were based on previous work of our group. Also, we detailed the proportion of trials coded with each rule to show their relative prevalence. Last, in ambiguous cases, trials were considered appropriate, so our work may have overestimated the proportion of trials with optimal post-progression treatment.

## Conclusion

We find that most anti-cancer RCTs do not report assessable post-progression data: 52% of FDA registration trials and 64% of top journal published trials. In 11.7% of FDA registration trials and 16.4% of published trials, post-progression treatment was reported and assessed as appropriate. In subgroup analysis of trials with assessable post-progression data with an OS advantage, fewer proportion of trials reported adequate post-progression therapy. Suboptimal post-progression treatment in the control arm may bias the results of an RCT beyond first PFS, particularly OS results. Regulatory rules should enforce higher requirements regarding post-progression treatment access and reporting.

## Supplementary Information

**Additional file 1: Method S1.** Published articles identification (p. 1). **Method S2.** FDA Registration Trials identification (p. 1). **Fig S1.** Schematic Illustration of The First Rule: Panel A (desirable): Optimal Post-Progression Therapy;  Panel B (suboptimal): Substandard Post-Progression Access To Preferred Therapy. (p. 2). **Fig S2.** Schematic Illustration of The Second Rule: Inappropriate Use of Crossover When The Experimental Drug Has No Proven Fundamental Efficacy. (p. 3). **Fig S3.** Schematic Illustration of The Third Rule: Panel A (desirable): High Proportion of Patient Receiving Post-Progression Treatment in Both Arms; Panel B (suboptimal): Low Proportion of Patients Receiving Post-Progression Treatment in Both Arms. (p. 4). **Table S1.** Tumor Types Of All Included Published Articles (*N* = 275) (p. 5). **Table S2.** Tumor Types Of All Included FDA Approvals (*N* = 77) (p. 6). **Table S3.** Trials With Assessable Post-Progression Data (*N*=104) Classified According To The Type Of Trials (assessing fundamental efficacy versus optimal sequencing) (p. 7). **Table S4.** Real-World Data With Tumor Type, Setting, Proportion Of Patients Having Access To Post-Progression Treatment, And References (when the setting is first line, post-progression treatment is the proportion of patients receiving a second line after receiving a first line) (p. 8). Supplement references (p. 8).

## Data Availability

All data on which this work was based are publicly available. The data generated during the study are available upon reasonable request from the corresponding author.

## Abbreviations

RCT: Randomized clinical trial
OS: Overall survival
PFS: Progression-free survival
FDA: United States Food and Drug Administration
SOC: Standard of care
