# Supplementary material for: Post-progression treatment in cancer randomized trials: a cross-sectional study of trials leading to FDA approval and published trials between 2018 and 2020
Source: BMC Cancer. 2023 May 17;23:448. doi: 10.1186/s12885-023-10917-z (PMC10189952; doi:10.1186/s12885-023-10917-z)
Supplement: Supplementary file 1 — Additional file 1: Method S1. Published articles identification (p. 1). Method S2. FDA Registration Trials identification (p. 1). Fig S1. Schematic Illustration of The First Rule: Panel A (desirable): Optimal Post-Progression Therapy; Panel B (suboptimal): Substandard Post-Progression Access To Preferred Therapy. (p. 2). Fig S2. Schematic Illustration of The Second Rule: Inappropriate Use of Crossover When The Experimental Drug Has No Proven Fundamental Efficacy. (p. 3). Fig S3. Schematic Illustration of The Third Rule: Panel A (desirable): High Proportion of Patient Receiving Post-Progression Treatment in Both Arms; Panel B (suboptimal): Low Proportion of Patients Receiving Post-Progression Treatment in Both Arms. (p. 4). Table S1. Tumor Types Of All Included Published Articles (N = 275) (p. 5). Table S2. Tumor Types Of All Included FDA Approvals (N = 77) (p. 6). Table S3. Trials With Assessable Post-Progression Data (N=104) Classified According To The Type Of Trials (assessing fundamental efficacy versus optimal sequencing) (p. 7). Table S4. Real-World Data With Tumor Type, Setting, Proportion Of Patients Having Access To Post-Progression Treatment, And References (when the setting is first line, post-progression treatment is the proportion of patients receiving a second line after receiving a first line) (p. 8). Supplement references (p. 8). [file 12885_2023_10917_MOESM1_ESM.docx]

**Supplement:**

Method S1: Published articles identification (p. 1)

Method S2: FDA Registration Trials identification (p. 1)

Fig S1: Schematic Illustration of The First Rule: Optimal Post-Progression Therapy, Panel A (desirable); Substandard Post-Progression Access To Preferred Therapy, Panel B (suboptimal). (p. 2)

Fig S2: Schematic Illustration of The Second Rule: Inappropriate Use of Crossover When The Experimental Drug Has No Proven Fundamental Efficacy. (p. 3)

Fig S3: Schematic Illustration of The Third Rule: High Proportion of Patient Receiving Post-Progression Treatment in Both Arms, Panel A (desirable); Low Proportion of Patients Receiving Post-Progression Treatment in Both Arms, Panel B (suboptimal). (p. 4)

Table S1: Tumor Types Of All Included Published Articles (N = 275) (p. 5)

Table S2: Tumor Types Of All Included FDA Approvals (N = 77) (p. 6)

Table S 3: Trials With Assessable Post-Progression Data (N=104) Classified According To The Type Of Trials (assessing fundamental efficacy versus optimal sequencing) (p. 7)

Table S4: Real-World Data With Tumor Type, Setting, Proportion Of Patients Having Access To Post-Progression Treatment, And References (when the setting is first line, post-progression treatment is the proportion of patients receiving a second line after receiving a first line) (p. 8)

Supplement references (p. 8)

Method S1: Published articles identification

We selected articles for this analysis from three top medical journals (The New England Journal of Medicine (NEJM), The Lancet, and JAMA) and three top cancer journals (The Lancet Oncology, Journal of Clinical Oncology (JCO), and JAMA Oncology) that publish clinical trial research (published between January 2018 and December 2020), as per impact-factor scores on Scimago Journal and Country Rank. For the published articles, we searched PubMed.gov selecting all articles that were published during the selected dates, limiting to studies with “randomized controlled trial" as the publication type. Retracted articles were excluded.

Method S2: FDA Registration Trials identification

We searched the FDA website (including FDA announcements) and a complete list of FDA approvals used previously for trials leading to a drug’s FDA approval (January 2018 and December 2020). For each approval we reviewed the official FDA initial announcement or label for trial data and then searched for the published trial (identification through NCT number).

Fig S1


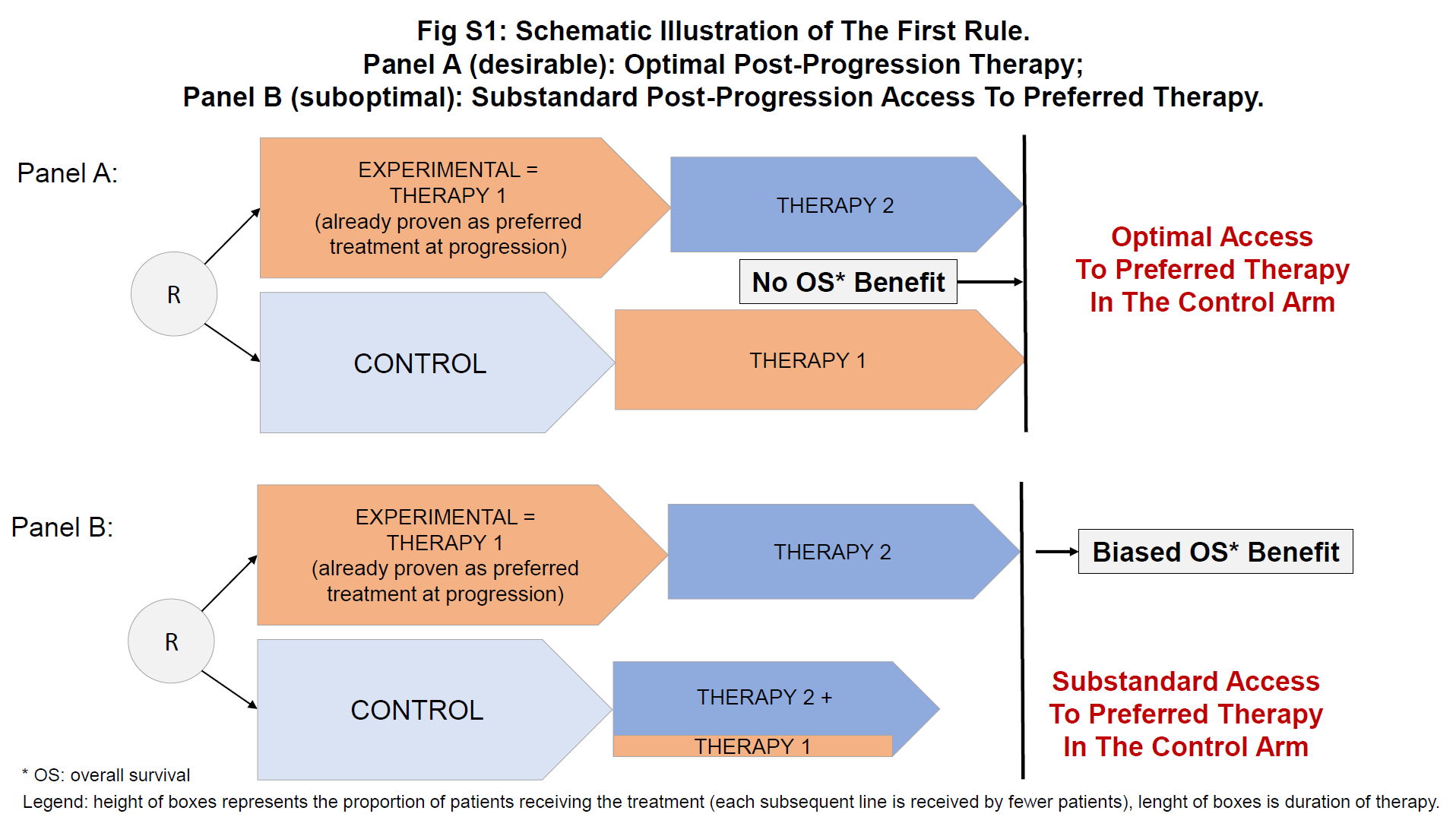


Fig S2


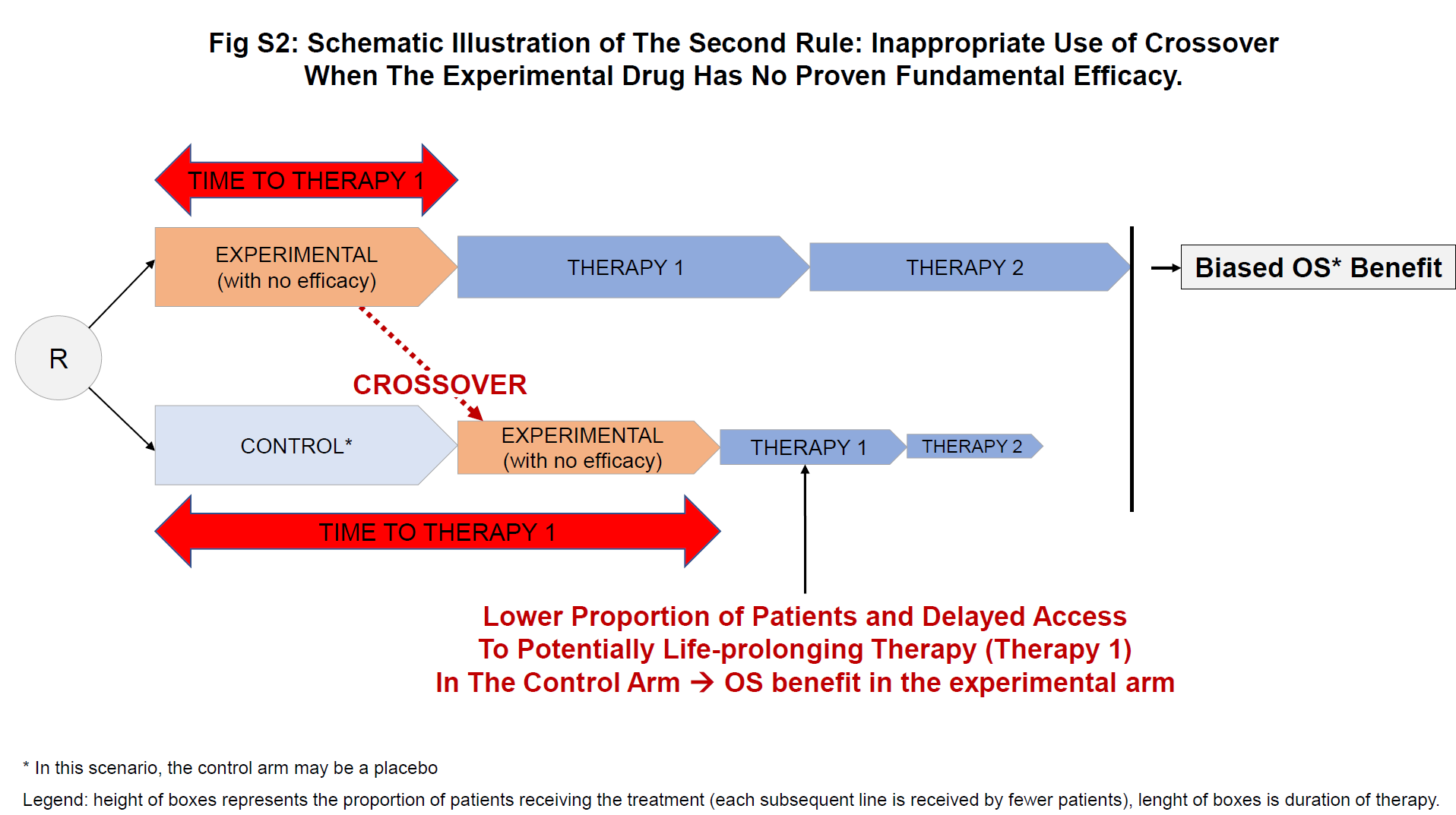


Fig S3


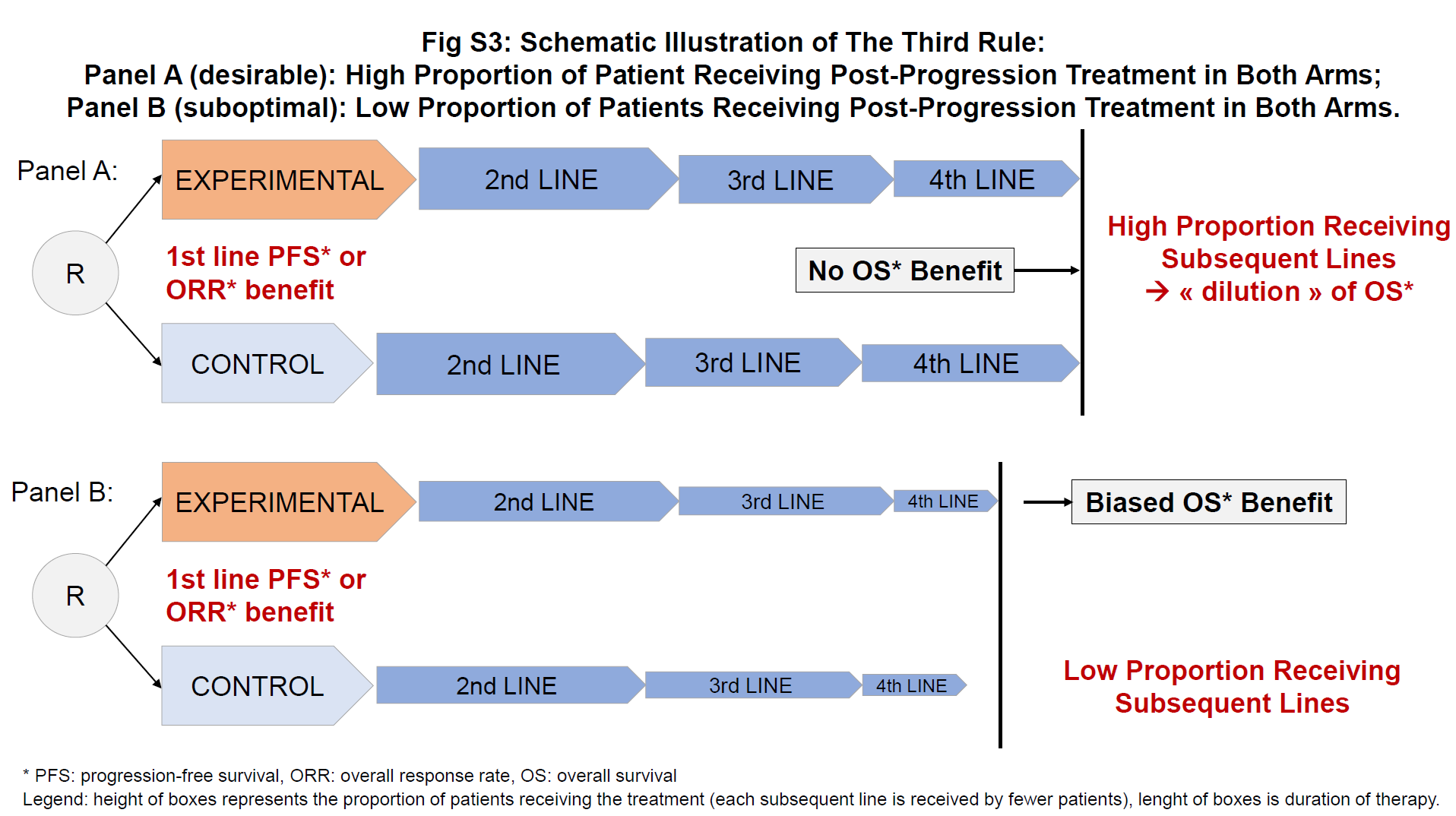


Table S1: Tumor Types Of All Included Published Articles (N = 275)

Table S2: Tumor Types Of All Included FDA Approvals (N = 77)

Table S3: Trials With Assessable Post-Progression Data (N=104) Classified According To The Type Of Trials (assessing fundamental efficacy *versus* optimal sequencing)

| Type of Trial | Testing  Fundamental Efficacy (N=49) | Testing  Optimal Sequence (N=55) | Overall (N=104) |
| --- | --- | --- | --- |
| Line | | | |
| First line | 18 (32.1%) | 38 (67.9%) | 56 |
| Maintenance | 1 (16.7%) | 5 (83.3%) | 6 |
| Mixed | 3 (75%) | 1 (25%) | 4 |
| Second or Subsequent | 22 (68.7%) | 10 (31.3%) | 32 |
| Third or Subsequent | 5 (83.3%) | 1 (16.7%) | 6 |
| Crossover | | | |
| Allowed | 3 (37.5%) | 5 (62.5%) | 8 |
| Not Allowed | 14 (73.7%) | 5 (26.3%) | 19 |
| Not Planned Nor Mentionned | 30 (42.3%) | 41 (57.7%) | 71 |
| Subsequently Allowed | 2 (40%) | 3 (60%) | 5 |
| Crossover as the trial design | 0 (0%) | 1 (100%) | 1 |
| Adequacy | | | |
| 1 - Limited access to a preferred option | 12 (35.3%) | 22 (64.7%) | 34* |
| 2 - Crossover to unproven therapy | 9 (100%) | 0 (0%) | 9* |
| 3 - Lower than real-life | 5 (38.5%) | 8 (61.5%) | 13* |
| 4 - Adequate | 23 (47.9%) | 25 (52.1%) | 48 |
| Positive overall survival results | | | |
| No | 29 (48.3%) | 31 (51.7%) | 60 |
| Yes | 20 (45.5%) | 24 (54.5%) | 44 |

* the total number of patients enrolled in trials with suboptimal post-progression therapy (corresponding to rule 1, 2 or 3) was n = 38 524.

Table S4: Real-World Data With Tumor Type, Setting, Proportion Of Patients Having Access To Post-Progression Treatment, And References (when the setting is first line, post-progression treatment is the proportion of patients receiving a second line after receiving a first line)

| Tumor Type | Setting | Post-Progression Treatment | References |
| --- | --- | --- | --- |
| Triple Negative Breast Cancer | First Line | 60% | ^1^ |
| Hormone Receptor Positive Breast Cancer | First Line | 80 to 93% | ^2^ |
| Non-Small Cell Lung Cancer | First Line | 53 % | ^3^ |
| Nonmetastatic castration-resistant Prostate Cancer | First Line | 97% | ^4^ |
| Small Cell Lung Cancer | First Line | 50 % | ^5^ |
| Colorectal Cancer | First Line | 74 | ^6^ |
| Gastric Cancer | First Line | 64% | ^7^ |
| Ovarian | Third Line | 67% | ^8^ |
| Metastatic Castration-Sensitive Prostate Cancer | First Line | 64% | ^9^ |
| Metastatic Castration-resistant Prostate Cancer | Second or subsequent | 42% | ^10^ |
| Papillary Renal | First Line | 72% | ^11^ |
| Sarcoma | First line | 60% | ^12^ |

References:

1. Skinner KE, Haiderali A, Huang M, Schwartzberg LS. Real-world effectiveness outcomes in patients diagnosed with metastatic triple-negative breast cancer. Future Oncol. mars 2021;17(8):931‑41.

2. Basile D, Gerratana L, Corvaja C, Pelizzari G, Franceschin G, Bertoli E, et al. First- and second-line treatment strategies for hormone-receptor (HR)-positive HER2-negative metastatic breast cancer: A real-world study. The Breast. 1 juin 2021;57:104‑12.

3. Davies J, Patel M, Gridelli C, Marinis F de, Waterkamp D, McCusker ME. Real-world treatment patterns for patients receiving second-line and third-line treatment for advanced non-small cell lung cancer: A systematic review of recently published studies. PLOS ONE. 14 avr 2017;12(4):e0175679.

4. Yokomizo A, Yonese J, Egawa S, Fukuhara H, Uemura H, Nishimura K, et al. Real-world use of enzalutamide in men with nonmetastatic castration-resistant prostate cancer in Japan. Int J Clin Oncol. 1 févr 2022;27(2):418‑26.

5. Steffens CC, Elender C, Hutzschenreuter U, Dille S, Binninger A, Spring L, et al. Treatment and outcome of 432 patients with extensive-stage small cell lung cancer in first, second and third line - Results from the prospective German TLK cohort study. Lung Cancer. avr 2019;130:216‑25.

6. Tampellini M, Di Maio M, Baratelli C, Anania L, Brizzi MP, Sonetto C, et al. Treatment of Patients With Metastatic Colorectal Cancer in a Real-World Scenario: Probability of Receiving Second and Further Lines of Therapy and Description of Clinical Benefit. Clin Colorectal Cancer. déc 2017;16(4):372‑6.

7. Cotes Sanchís A, Gallego J, Hernandez R, Arrazubi V, Custodio A, Cano JM, et al. Second-line treatment in advanced gastric cancer: Data from the Spanish AGAMENON registry. PLoS One. 31 juill 2020;15(7):e0235848.

8. Beachler DC, Lamy FX, Russo L, Taylor DH, Dinh J, Yin R, et al. A real-world study on characteristics, treatments and outcomes in US patients with advanced stage ovarian cancer. J Ovarian Res. déc 2020;13(1):1‑13.

9. Freedland SJ, Sandin R, Sah J, Emir B, Mu Q, Ratiu A, et al. Treatment patterns and survival in metastatic castration-sensitive prostate cancer in the US Veterans Health Administration. Cancer Medicine. 2021;10(23):8570‑80.

10. de Wit R, Freedland SJ, Oudard S, Marinov G, Capart P, Combest AJ, et al. Real-world evidence of patients with metastatic castration-resistant prostate cancer treated with cabazitaxel: comparison with the randomized clinical study CARD. Prostate Cancer Prostatic Dis. 17 janv 2022;1‑7.

11. Staehler M, Goebell PJ, Müller L, Emde TO, Wetzel N, Kruggel L, et al. Rare patients in routine care: Treatment and outcome in advanced papillary renal cell carcinoma in the prospective German clinical RCC-Registry. International Journal of Cancer. 2020;146(5):1307‑15.

12. Savina M, Le Cesne A, Blay JY, Ray-Coquard I, Mir O, Toulmonde M, et al. Patterns of care and outcomes of patients with METAstatic soft tissue SARComa in a real-life setting: the METASARC observational study. BMC Medicine. 10 avr 2017;15(1):78.
